# Blood Glucose Prediction with Variance Estimation Using Recurrent Neural Networks

**DOI:** 10.1007/s41666-019-00059-y

**Published:** 2019-12-01

**Authors:** John Martinsson, Alexander Schliep, Björn Eliasson, Olof Mogren

**Affiliations:** 1 RISE Research Institutes of Sweden, Gothenburg, Sweden; 2 Gothenburg University, Gothenburg, Sweden; 3 Sahlgrenska University Hospital, Gothenburg, Sweden

**Keywords:** Recurrent neural networks, Blood glucose prediction, Type 1 diabetes

## Abstract

Many factors affect blood glucose levels in type 1 diabetics, several of which vary largely both in magnitude and delay of the effect. Modern rapid-acting insulins generally have a peak time after 60–90 min, while carbohydrate intake can affect blood glucose levels more rapidly for high glycemic index foods, or slower for other carbohydrate sources. It is important to have good estimates of the development of glucose levels in the near future both for diabetic patients managing their insulin distribution manually, as well as for closed-loop systems making decisions about the distribution. Modern continuous glucose monitoring systems provide excellent sources of data to train machine learning models to predict future glucose levels. In this paper, we present an approach for predicting blood glucose levels for diabetics up to 1 h into the future. The approach is based on recurrent neural networks trained in an end-to-end fashion, requiring nothing but the glucose level history for the patient. Our approach obtains results that are comparable to the state of the art on the Ohio T1DM dataset for blood glucose level prediction. In addition to predicting the future glucose value, our model provides an estimate of its certainty, helping users to interpret the predicted levels. This is realized by training the recurrent neural network to parameterize a univariate Gaussian distribution over the output. The approach needs no feature engineering or data preprocessing and is computationally inexpensive. We evaluate our method using the standard root-mean-squared error (RMSE) metric, along with a blood glucose-specific metric called the surveillance error grid (SEG). We further study the properties of the distribution that is learned by the model, using experiments that determine the nature of the certainty estimate that the model is able to capture.

## Introduction

Our future will be recorded and quantified in unprecedented temporal resolution. A rapidly increasing variety of variables gets stored, describing activities we engage in as well as physiological and medical phenomena. One example is the increasingly wide adoption of continuous blood glucose monitoring systems (CGM) which has given type 1 diabetics (T1D) a valuable tool for closely monitoring and reacting to their current blood glucose levels and trends. CGM data helps patients manage their insulin distribution by providing an informative source of data to act upon. CGM availability has also been of crucial importance for the development and use of closed-loop systems such as OpenAPS [15]. Blood glucose levels adhere to complex dynamics that depend on many different variables (such as carbohydrate intake, recent insulin injections, physical activity, stress levels, the presence of an infection in the body, sleeping patterns, hormonal patterns, etc) [4, 9]. This makes predicting the short-term blood glucose changes (up to a few hours) a challenging task, and developing machine learning (ML) approaches an obvious approach for improving patient care. However, acquiring domain expertise, understanding sensors, and hand-crafting features is expensive and not easy to scale up to further applications. Sometimes natural, obviously important and well-studied variables (e.g., caloric intake for diabetics) might be too inconvenient to measure for end-users. On the other hand, deep learning approaches are a step towards automated machine learning, as features, classifiers and predictors are simultaneously learned. Thus, they present a possibly more scalable solution to the myriad of machine learning problems in precision health management resulting from technology changes alone.

In this paper, we present a neural network model trained to predict blood glucose levels from CGM history, and demonstrate that:

- It is feasible to predict future glucose levels from glucose levels alone.
- Appropriate models can be trained by non-experts without feature engineering or complicated training procedures.
- The proposed model can quantify the uncertainty in its predictions to alert users to the need for extra caution or additional input.

Our method was trained and evaluated on the Ohio T1DM dataset for blood glucose level prediction (see [16] for details).

## Modeling Blood Glucose Levels Using Recurrent Neural Networks

A recurrent neural network (RNN) is a feed-forward artificial neural network that can model a sequence of arbitrary length, using weight sharing between each position in the sequence. In the basic RNN variant, the transition function at time *t* is a linear transformation of the hidden state *h*_*t*− 1_ and the input, followed by a point-wise non-linearity:

$$ \mathbf{h}_{t} = \tanh(W \mathbf{x}_{t} + U \mathbf{h}_{t-1} + \mathbf{b}),$$

where *W* and *U* are weight matrices, **b** is a bias vector, and tanh is the selected nonlinearity. *W*, *U*, and **b** are typically trained using some variant of stochastic gradient descent (SGD).

Basic RNNs struggle with learning long-range dependencies and suffer from the vanishing gradient problem. This makes them difficult to train [1, 12], and has motivated the development of the long short-term memory (LSTM) architecture [13], that to some extent solves these shortcomings. An LSTM is an RNN where the cell at each step *t* contains an internal memory vector **c**_*t*_, and three gates controlling what parts of the internal memory will be kept (the forget gate **f**_*t*_), what parts of the input that will be stored in the internal memory (the input gate **i**_*t*_), as well as what will be included in the output (the output gate **o**_*t*_). In essence, this means that the following expressions are evaluated at each step in the sequence, to compute the new internal memory **c**_*t*_ and the cell output **h**_*t*_. Here “⊙” represents element-wise multiplication and *σ*(⋅) is a logistic sigmoid function.

$$ \begin{array}{@{}rcl@{}} \mathbf{i}_{t} &=& \sigma(W_{i} \mathbf{x}_{t} + U_{i} \mathbf{h}_{t-1} + \mathbf{b}_{i}), \\ \mathbf{f}_{t} &=& \sigma(W_{f} \mathbf{x}_{t} + U_{f} \mathbf{h}_{t-1} + \mathbf{b}_{f}),\\ \mathbf{o}_{t} &=& \sigma(W_{o} \mathbf{x}_{t} + U_{o} \mathbf{h}_{t-1} + \mathbf{b}_{o}),\\ \mathbf{u}_{t} &=& \tanh(W_{u} \mathbf{x}_{t} + U_{u} \mathbf{h}_{t-1} + \mathbf{b}_{u}),\\ \mathbf{c}_{t} &=& \mathbf{i}_{t} \odot \mathbf{u}_{t} + \mathbf{f}_{t} \odot \mathbf{c}_{t-1},\\ \mathbf{h}_{t} &=& \mathbf{o}_{t} \odot \tanh(\mathbf{c}_{t}). \end{array} $$

**Fig. 1:**
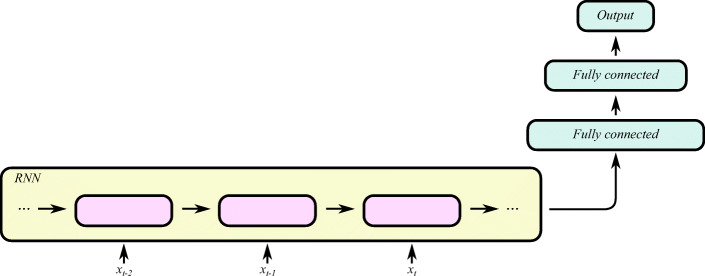
High-level illustration of the RNN model used in this work. Each RNN cell processes the blood glucose level at one time step, and at prediction time *t*, the RNN output *h*_*t*_ is used as input to a stack of fully connected layers finally outputting the parameters for the predicted distribution of the future glucose level. Boxes represent neural network layers (processing), and each arrow represents a vector fed from a layer to the next

We model the blood glucose levels using a recurrent neural network (see Fig. 1), working on the sequence of input data provided by the CGM sensor system. The network consists of LSTM cells. The whole model takes as input a stream of blood glucose measurements from the CGM system and outputs one prediction regarding the blood glucose level after time *T* (we present experimental evaluation for *T* ∈{30,60} min). An RNN is designed to take a vector of inputs at each time step, but in the case of feeding the network with blood glucose measurements only, the input vectors are one dimensional (effectively scalar valued).

The output vector from the final LSTM cell (see **h**_*t*_ in Fig. 1) in the sequence is fed through a fully connected neural network with two hidden dense layers and one output layer. The hidden layers consist of 512 and 256 neurons respectively, with rectified linear activations and a dropout of 20% and 30% respectively. The dropout layers mitigate over-fitting the model to the training data. The output layer consists of two neurons: one with a linear activation and one with an exponential activation.

The output is modeled as a univariate Gaussian distribution [3], using one value for the mean, *μ*, and one value for the standard deviation, *σ*. This gives us an estimate of the confidence in the models’ predictions.

1 $$ \mu = W_{1} \mathbf{h}_{fc} + \mathbf{b}_{1}  $$

2 $$ \sigma = \exp(W_{2} \mathbf{h}_{fc} + \mathbf{b}_{2})  $$

where **h**_*fc*_ is the output of the last hidden dense layer. As in [3], we use a linear activation for the mean (see (1)), and an exponential activation for the standard deviation (see (2)) to ensure that the output is positive since standard deviation is not defined for negative values.

The negative log-likelihood (NLL) loss function is derived from the Gaussian probability density function,

$$\mathcal{L} =\frac{1}{k}\sum\limits_{i=0}^{k}-\log\left( \mathcal{N}(y_{i} | \mu_{i}, {\sigma^{2}_{i}})\right),$$

where *y*_*i*_ is the target value from the data and *μ*_*i*_ and *σ*_*i*_ are the network’s output given the input sequence ***x***_*i*_. This way of modeling the prediction facilitates basing decisions on the predictions, by providing an estimate of the prediction uncertainty.

### Physiological Loss Function

We also trained the model with a glucose-specific loss function [10], which is a metric that combines the mean squared error with a penalty term for predictions that would lead to contraindicated interventions possibly leading to clinically critical situations.

### Preliminary Study

Preliminary results from this study were presented at *The 3rd international workshop on knowledge discovery in healthcare data* at ICML/IJCAI 2018 [17]. However, since the preliminary workshop paper, the proposed model has been further refined by a more thorough exploration of hyperparameters and changes to the model design (such as the activation functions), and the results have consequently improved. This paper also includes a more thorough analysis, such as surveillance error grid analysis and an investigation of the variance predictions using controlled synthetic data. The model in the current study is trained on all available training data whereas the preliminary study considered models trained specifically for one patient at a time.

### Experimental Setup

We trained and evaluated our method on the Ohio T1DM dataset for blood glucose level prediction [16]. The data consists of blood glucose level measurements for six people with type 1 diabetes (T1D). A continuous glucose monitoring (CGM) device was used to collect eight weeks of data, at 5-min intervals, for each of the six patients. There were two male patients and four female patients between 40 and 60 years old. All patients were on insulin pump therapy. There are roughly the same number of blood glucose level observations for each patient in the training and testing data (see Table 1). The patients have been de-identified and are referred to by ID numbers. Patients 563 and 570 were male, and patients 559, 575, 588, and 591 were female.

**Table 1:** The number of blood glucose level measurements that are used as training and testing data for each patient in the Ohio T1DM dataset for blood glucose level prediction

Patient ID	Training examples	Test examples	Gender
559	10796	2514	F
563	12124	2570	M
570	10982	2745	M
575	11866	2590	F
588	12640	2791	F
591	10847	2760	F

The table also shows the gender for each patient

There are other data self-reported by the patients such as meal times with carbohydrate estimates; times of exercise, sleep, work, stress, and illness; and measures of heart rate, galvanic skin response, skin temperature, air temperature, and step count. In this work, we consider the problem of predicting future blood glucose levels using only previous blood glucose level measurements. The only preprocessing done on the glucose values is scaling by 0.01 as in [19] to get the glucose values into a range suitable for training.

#### Dataset Split

For all patients, we take the first 60% of the data and combine it into a training set, we take the following 20% of the data and combine it into a validation dataset used for early stopping, and we choose the hyperparameters by the root-mean-squared error performance on the last 20% of the data.

#### Hyperparameter Selection

**Fig. 2:**
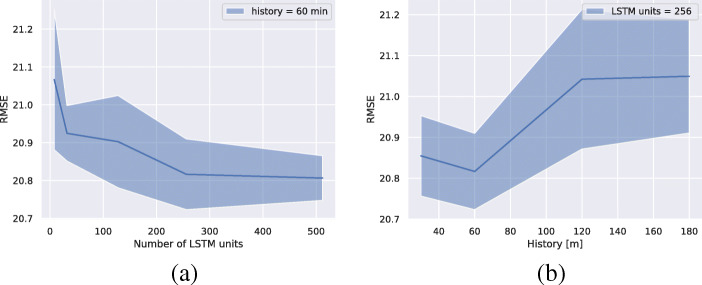
Mean RMSE and standard deviation (shaded region) for the validation data over 30 different random initializations for each hyperparameter configuration. A history of 60 min means that the LSTM use the blood glucose measurements taken during the last 60 min to make a prediction 30 min into the future

The hyperparameters for the model are chosen using a grid search over different parameter configurations. The size of the LSTM state was selected from the range {8,32,128,256,512} and the amount of history from {30,60,120,180} min. We use Adam optimizer with a batch size of 1024 and a learning rate of 10^− 3^ and set the early stopping criterion to 20 epochs. That is, if no improvement is observed on the validation data for the last 20 epochs, we terminate the training. For each hyperparameter configuration, we train with 30 different random seeds and choose a model configuration with a low mean RMSE score while keeping the model complexity low. The results are shown in Fig. 2. Using a glucose level history of 60 min to make a prediction results in the lowest RMSE on the validation data. The difference in RMSE between using 256 and 512 LSTM units is very small, and we choose 256 LSTM units to keep the model complexity low.

We then choose the learning rate and the batch size by fixing the number of LSTM units and the amount of history used and instead vary the learning rate between 10^− 3^ and 10^− 5^ and the batch size between 128 and 1024. The converged models give approximately the same validation loss for different learning rates and batch size, but a learning rate of 10^− 3^ and a batch size of 1024 leads to faster convergence and is therefore chosen.

#### Final Models

The final models were trained using 60 min of glucose level history for predictions 30 and 60 min into the future. The setup for the final training was to train on the first 80*%* of the glucose level training data from all patients, and do early stopping on the last 20*%*. The final models were trained with Adam optimizer with a learning rate of 10^− 3^, a batch size of 1024, a maximum of 10,000 epochs, and an early stopping criterion set to 200 epochs. We train 100 models with different random initializations of the parameters and report the mean evaluation score for all 100 models on the test data. A link to the source code of the model and the training scripts has been provided in Appendix A.

#### Evaluation

The final models were evaluated on the officially provided test partition of the dataset. Root-mean-squared error (RMSE) and surveillance error scores are reported. Each CGM value in the test set is considered a prediction target provided that it is preceded by enough CGM history. The number of missing predictions depends on the number of gaps in the data, i.e., the number of pair-wise consecutive measurements in the glucose level data where the time step is not exactly five minutes. We do not interpolate or extrapolate to fill the missing values since it is unclear how much bias this would introduce, but instead only use data for which it is possible to create the (*x*, *y*) pairs with a given glucose history, *x*, and regression target, *y*, for a given prediction horizon. As a result, we make predictions for approximately 90% of the test data. The discarded test-points are not counted in the evaluation.

#### Computational Requirements

In our experimental setup, training of the model could be performed on a commodity laptop. The model is small enough to fit in the memory of, and be used on mobile devices (e.g., mobile phones, blood glucose monitoring devices). Training could initially be performed offline and then incremental training would be light enough to allow for training either on the devices or offline.

## Results

The results presented in Table 2 are the mean RMSE and the standard deviation on the test data for 100 models with the same hyperparameter configuration but with different random initializations presented for each patient individually and as a mean over all patients. The baseline, *t*_0_, is just naively predicting the last known glucose value.

**Table 2:** Mean and standard deviation of the root-mean-squared error (RMSE) per patient over 100 different random initializations and the mean over all patients for predicting glucose levels 30 respectively 60 min into the future

	30-min horizon		60-min horizon	
Patient ID	LSTM	*t*_0_	LSTM	*t*_0_
559	18.773 ± 0.179	23.401	33.696 ± 0.365	39.404
570	15.959 ± 0.374	18.809	28.468 ± 0.834	31.577
588	18.538 ± 0.106	21.893	31.337 ± 0.210	35.928
563	17.961 ± 0.192	20.786	29.012 ± 0.169	34.032
575	21.675 ± 0.218	25.452	33.823 ± 0.268	39.164
591	20.294 ± 0.107	24.249	32.083 ± 0.182	38.219
*μ*	18.867	22.432	31.403	36.387
*σ*	± 1.794	± 2.217	± 2.078	± 2.860

*t*_0_ denotes the naive baseline of predicting the last value

The glucose level of patient 575 is harder to predict than the glucose level for patient 570, as seen in Table 2 where the mean RMSE for patient 570 is 15.959 and the mean RMSE for patient 575 is 21.675. We observe that patient 575 has higher glucose variability than patient 570. The percentage of first differences greater than 10 mg/dl/5m or lower than − 10 mg/dl/5m are 7.3% for patient 575 and 3.0% for patient 570 in the test data. Abnormal rates of change are potentially harder to predict, which may partially explain why the performance is lower on patient 575 than on patient 570.

**Fig. 3:**
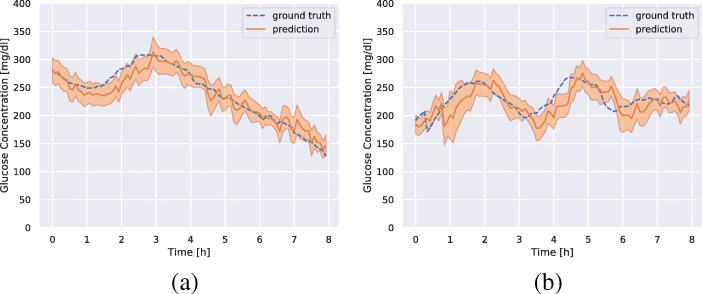
Prediction (orange), predicted standard deviation (shaded orange), and the ground truth glucose concentration (dashed blue) for patients 570 (**a**) and 575 (**b**). The plot shows 8 h of predictions starting from an arbitrarily chosen time for each patient in the test data. The predictions are 30 min into the future

Figure 3a and b show the predicted glucose concentrations and the corresponding ground truth glucose concentrations for patient 570 and 575. We see that the predictions follow the ground truth well in most regions, but that there is a lag in the predicted values for quickly increasing regions.

### Surveillance Error Grid

In addition to the RMSE metric, it is informative to know how well the model performs in a clinical scenario. We therefore use the surveillance error grid [14] to define an evaluation criterion that accounts for the clinical risk of making an incorrect prediction. The criterion is defined by a bilinear interpolation of the 600 × 600 surveillance error grid and is denoted by $e(y,\hat {y}) \in [0, 4]$, where $e(y, \hat {y})$ is the estimated clinical risk of predicting the blood glucose concentration $\hat {y}\in [0, 600]$ (in mg/dl) given that *y* ∈ [0,600] is the ground truth concentration. Let $\{\hat {y}_{t} | t\in \{1,\dots , T\}\}$ be the predictions for a patient at each discrete time step *t*, and let $\{y_{t} | t\in \{1,\dots , T\}\}$ be the corresponding ground truth reference concentrations. The criterion is then given by

$$ SE = \frac{1}{T}\sum\limits_{t=1}^{T} e(y_{t}, \hat{y}_{t}). $$

Note that the criterion is only defined for blood glucose concentrations up to 600 mg/dl, which is the limit of most CGMs and any model that predicts values outside of this region should be discarded or constrained.

In Table 3, we present the mean surveillance error and the standard deviation on the test data for the 100 different random seeds for each patient individually and a mean and standard deviation for all patients. We can see that the performance is worse for patient 575 than for patient 570, but according to this metric the model performs worst on patient 591.

**Table 3:** Results individually per patient and averages in predicting glucose levels with a 30- and 60-min prediction horizon respectively

	30-min horizon		60-min horizon	
Patient ID	LSTM	*t*_0_	LSTM	*t*_0_
559	0.178 ± 0.003	0.224	0.331 ± 0.003	0.386
570	0.105 ± 0.002	0.141	0.195 ± 0.004	0.244
588	0.177 ± 0.002	0.214	0.291 ± 0.002	0.349
563	0.176 ± 0.002	0.222	0.293 ± 0.002	0.360
575	0.224 ± 0.004	0.272	0.389 ± 0.005	0.434
591	0.256 ± 0.003	0.299	0.396 ± 0.003	0.478
*μ*	0.186	0.229	0.316	0.375
*σ*	± 0.047	± 0.050	± 0.068	± 0.073

The table shows the surveillance error (SE) of the LSTM model trained with NLL. *t*_0_ refers to the naive baseline of predicting the last value

**Fig. 4:**
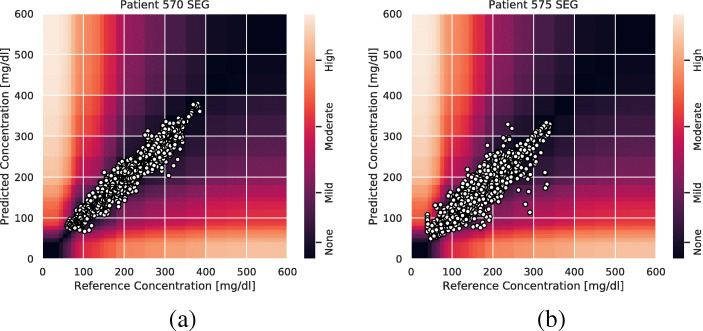
The surveillance error grid overlayed with each model prediction concentration and reference concentration for patient 570 (**a**) and patient 575 (**b**). The predictions are for all the test data points preceded by 90 min of consecutive glucose level measurements without missing values. That is, 60 min of history and a 30-min prediction horizon. The predicted concentrations and the corresponding reference concentrations are illustrated with white circles, and the estimated risk of a predicted concentration given the ground truth reference concentration is illustrated by color in the plot. The risk zones are divided into four main risk categories: none, mild, moderate, and high

In Fig. 4, we see that the predictions for patient 570 are mostly concentrated to the none and mild risk regions, but for patient 575 we can see that there are a few predictions in the moderate to high-risk regions as well. Additional figures for the other patients are provided in Appendix B.

### Noise Experiments

**Fig. 5:**
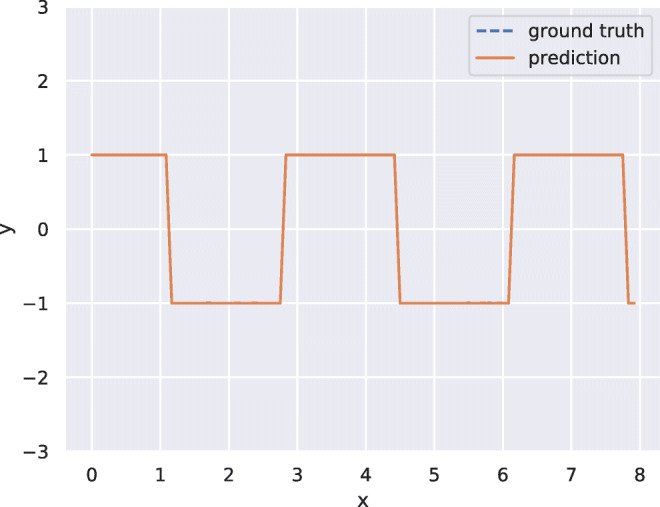
The predictions from the proposed model trained on a deterministic squared waveform with step length 20 and states in − 1 and 1. The predicted mean is plotted in orange and the predicted standard deviation is plotted in shaded orange. The signal we train on is plotted in blue. The ground truth signal is not visible in the plot since the model solves the problem and the predictions occlude the ground truth

**Fig. 6:**
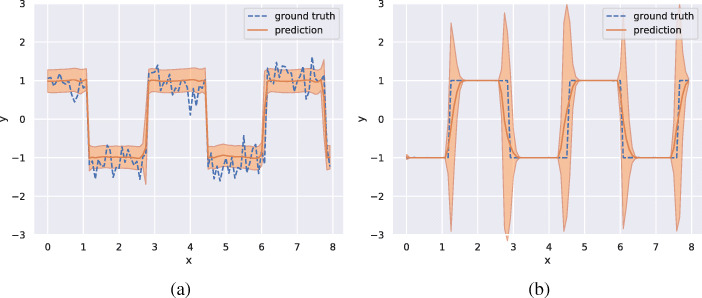
The predictions from the proposed model trained on a waveform signal with a step length of 20 and states − 1 and 1 with an added noise drawn form a normal distribution with mean 0 and standard deviation 0.3 (**a**), and a waveform with a step length of 20 with an added noise to the step length drawn from a normal distribution with mean zero and standard deviation 3.0 (**b**). The predicted mean is plotted in orange and the predicted standard deviation is plotted in shaded orange. The signal we train on is plotted in blue

To get insight into what uncertainty the model is able to learn, we have conducted three experiments to isolate different types of noise added to a deterministic signal. The deterministic signal is a simple squared waveform with a step length of 20 and two state values of − 1 and 1 (see Fig. 5). We add two types of noise which we will call measurement noise and state length noise. The measurement noise is drawn from a normal distribution with a zero mean and a standard deviation of 0.3 and is simply added to the state value (see Fig. 6a). The state length noise is drawn from a normal distribution with a zero mean and a standard deviation of 3 and is added to the step length of the waveform, i.e., the length we stay in each state is normally distributed with a mean of 20 and a standard deviation of 3 (see Fig. 6b). The experiment with measurement noise indicates that the model learns to attribute a higher uncertainty to the prediction, when the CGM is giving readings with higher noise levels. The experiment with noisy state length is set up in such a way that the model can not know when the state change will occur, and that this uncertainty gets higher the longer we have stayed in a state. We can see that the model learns to attribute high uncertainty to predictions that are made close to a state change.

## Discussion

In this paper, we have proposed a recurrent neural network model that can predict blood glucose levels in type 1 diabetes for horizons of up to 60 min into the future using only blood glucose level as inputs. We achieve results comparable to state-of-the-art methods on the standard Ohio T1DM dataset for blood glucose level prediction.

### End-to-End Learning

Our results suggest that end-to-end machine learning is feasible for precision health management. This allows the system to learn all internal representations of the data, and reduces the human effort involved—avoiding labor-intensive prior work by experts hand-crafting features based on extensive domain knowledge.

### Quantifying Uncertainty

Our model gives an estimate of the standard deviation of the prediction. This is a useful aspect for a system which will be used by CGM users for making decisions about the administration of insulin and/or caloric intake. The predicted standard deviation can also be a useful signal for downstream components in a closed-loop system, making automatic decisions for a patient. The results in Fig. 3 show the predicted standard deviation for patient 570 and patient 575, the ones where the model is the most and the least successful in prediction accuracy, respectively. One principal problem is that disambiguating between intra-patient variation and sensor errors is unlikely to be feasible.

### Physiological Loss Function

To our surprise, we did not see improvements when using a physiologically motivated loss function [10] for training (results not shown). This is essentially a smoothed version of the Clarke error grid [7]. Of course, our findings are not proof that such loss functions cannot improve results. Possibly a larger scale investigation, exploring in particular a larger area of the parameter space and different training regimes, might provide further insights. Penalizing errors for hypo- or hyper-glycemic states should lead to better real-world performance, as we observed comparatively larger deviations in minima and maxima. One explanation for that is the relative class imbalance, as extrema are rare. This could be countered with data augmentation techniques.

### Model Selection

Even though the different patients pose varying challenges for the prediction task (see Fig. 2), we obtain the best result when training our model on the training data from all patients at once. This suggests that there are patterns governing blood glucose variability that can generalize between different patients, and that the model benefits from having access to more data.

### Missing Data

There are gaps in the training data with missing values. Most of the gaps are less than 10 h, but some of the gaps are more than 24 h. The number of missing data points account for roughly 23 out of 263 days of the total amount of patient data or 9% of the data. The gaps could be filled using interpolation, but it is not immediately clear how this would affect either the training of the models, or the evaluation of the models since this would introduce artificial values. Filling a gap of 24 h using interpolation would not result in realistic data. Instead, we have chosen not to fill the gaps with artificial values and limit our models to be trained and evaluated only on real data. This has its own limitations since we can only consider prediction targets with enough glucose level history, and therefore, not predict the initial values after a gap, but the advantage is that model training and evaluation is not biased by the introduction of artificial values.

### Additional Patient Data

As mentioned in the description of the dataset, there are other data self-reported by the patients such as meal times with carbohydrate estimates, times of exercise, sleep, work, stress, and illness; and measures of heart rate, galvanic skin response, skin temperature, air temperature and step count. From the results in this work, we conclude that a simple setup using only CGM history obtains results that are on par with more complex solutions that do incorporate more features. It is well documented that the additional features do affect blood glucose dynamics but the dependencies may be more subtle and complex and thus harder to learn. This motivates further work to develop models that can leverage the additional information and make more accurate predictions.

## Related Work

Early work on predicting blood glucose levels from CGM data include Bremer, et al. [4], who explored the predictability of data from CGM systems, and showed how you can make predictions based on autocorrelation functions. Sparacino, et al. [23] proposed a first-order auto-regressive model.

Wiley [24] proposed using support vector regression (SVR) to predict blood sugar levels from CGM data. They report RMSE of 4.5 mg/dl, but this is using data that was aggressively smoothed using a regularized cubic spline interpolation. Bunescu, et al. [5] extended this work with physiological models for meal absorption dynamics, insulin dynamics, and glucose dynamics to predict blood glucose levels 30 and 60 min into the future. They obtained a relative improvement of about 12% in prediction accuracy over the model proposed by Wiley. The experiments in [5] is performed on non-smoothed data.

There have been approaches using neural networks to predict blood glucose levels. Perez, et al. [22] presented a feed-forward neural network (FFNN) taking CGM history as input, and predicting the level 15, 30, and 45 min into the future. RMSE accuracy for 30-min predictions is similar to those of [24]. Mougiakakou et al. [20] showed that RNNs can be used to predict blood glucose levels from CGM data. They evaluated their method on four different children with type 1 diabetes, and got some promising results. On average, they reported an RMSE accuracy of 24.1 mg/dl.

Some papers have incorporated additional information (e.g., carbohydrate/meal intake, insulin injections, etc). Pappada et al. [21] proposed an FFNN taking as input CGM levels, insulin dosages, metered glucose levels, nutritional intake, lifestyle, and emotional factors. Despite having all this data at its disposal, the model makes predictions 75 min into the future with an RMSE score of 43.9 mg/dl. Zecchin et al. [26] proposed a neural network approach in combination with a first-order polynomial extrapolation algorithm to produce short-term predictions on blood glucose levels, taking into account meal intake information. The approach is evaluated both on simulated data, and on real data from 9 patients with Abbott FreeStyle Navigator. None of the above-mentioned approaches have the ability to output a confidence interval.

A problem when modeling continuous outputs trained using least squares as a training criterion is that the model tends to learn a conditional average of the targets. Modeling a distribution over the outputs may limit this problem and make training more stable. Mixture density networks were proposed by [3]. By allowing the output vector from a neural network model to parameterize a mixture of Gaussians, they manage to learn a mapping even when the targets are not unique. Besides enabling learning stability, this also allows the model to visualize the certainty of its predictions. A similar approach was used together with RNNs in [11], to predict the distribution of next position for a pen during handwriting.

The release of the Ohio dataset [16] in combination with *The blood glucose level prediction challenge (BGLP)* at *The workshop on knowledge discovery in healthcare data (KDH)* 2018, spurred further interest on blood glucose prediction models. At the workshop, a preliminary version of this study was presented [17]. While a challenge was formulated, no clear winner could be decided, because of differences in the evaluation procedure. The results listed below cannot directly be compared to the results in this paper due to these differences. However, they all refer to predictions made with a 30-min horizon. While our study has focused on predicting the blood glucose levels using only the CGM history as input, all methods below use more features provided in the dataset such as carbohydrate intake and insulin distribution, and none of them gives an estimate of the uncertainty.

Chen et al. [6] used a recurrent neural network with dilations to model the data. Dilations allow a network to learn hierarchical structures and the authors chose to use the CGM values, insulin doses, and carbohydrate intake from the data, resulting in an average RMSE of 19.04 mg/dl. Xie et al. [25] compared autoregression with exogeneous inputs (ARX) with RNNs and convolutional neural networks (CNNs), and concluded that the simpler ARX models achieved the best scores on the Ohio blood glucose data, with an average RMSE of 19.59 mg/dl. Contreras et al. [8] used grammatical evolution (GE) in combination with feature engineering to search for a predictive model, obtaining an average RMSE of 24.83 mg/dl. Bertachi et al. [2] reported an average RMSE of 19.33 mg/dl by using physiological models for insulin onboard, carbohydrates onboard, and activity onboard, which are fed as features to a feed-forward neural network. Midroni et al. [18] employed XGBoost with a thorough investigation of feature importance and reported an average RMSE of 19.32 mg/dl. Zhu et al. [27] trained a CNN with CGM data, carbohydrate intake, and insulin distribution used as features and obtained an average RMSE of 21.72 mg/dl.

## Conclusions

In this paper, we presented a deep neural network model that learns to predict blood glucose levels up to 60 min into the future. The model parameterize a univariate Gaussian output distribution, facilitating an estimate of uncertainty in the prediction. Our results make a clear improvement over the baseline, and motivate future work in this direction.

However, it is clear that the field is in desperate need of larger data sets and standards for the evaluation. Crowd sourcing from patient associations would be one possibility, but differences in sensor types and sensor revisions, life styles, and genetic mark-up are all obvious confounding factors. Understanding sensor errors by measuring glucose level in vivo, for example in diabetes animal models, with several sensors simultaneously would be very insightful, and likely improve prediction quality. Another question concerns preprocessing in the sensors, which might be another confounding factor in the prediction. While protection of proprietary intellectual property is necessary, there has been examples, e.g., DNA microarray technology, where only a completely open analysis process from the initial steps usually performed with vendor’s software tools to the final result helped to realize the full potential of the technology.

## Figures and Tables

**Fig. 7:**
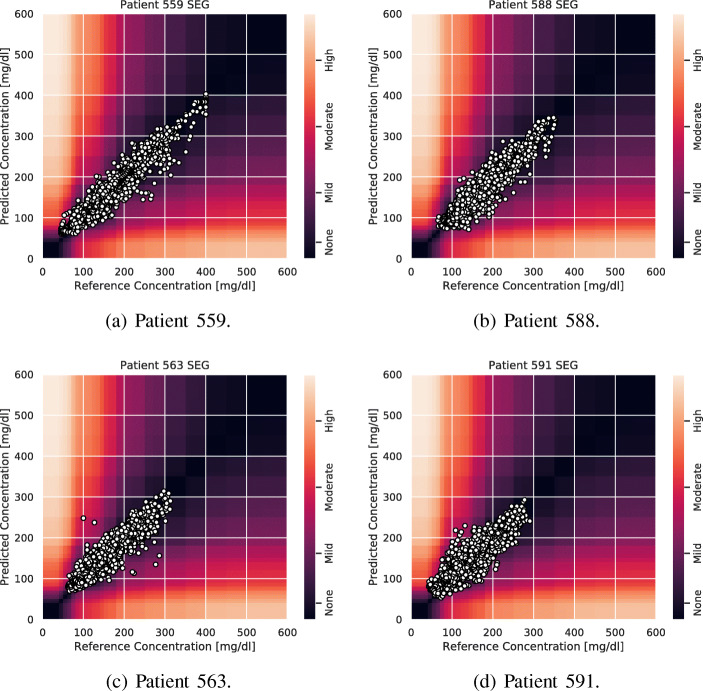
The surveillance error grid overlayed with each model prediction concentration and reference concentration for all patients. The predicted concentrations and the corresponding reference concentrations are illustrated with white circles, and the estimated risk of a predicted concentration given the ground truth reference concentration is illustrated by color in the plot. The risk zones are divided into four main risk categories: none, mild, moderate and high

**Fig. 8:**
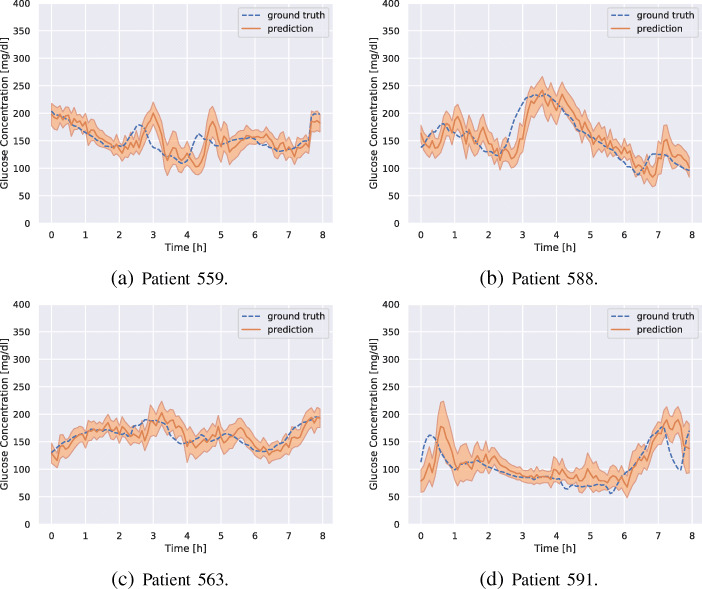
Prediction (orange), the predicted standard deviation (shaded orange), and the ground truth glucose concentration (dashed blue) for all patients. The plot shows 8 h of predictions starting from an arbitrarily chosen time for each patient. The predictions are 30 min into the future

## Appendix: A. Software

The software including all scripts to reproduce the computational experiments is released under an open-source license and available from https://github.com/johnmartinsson/blood-glucose-prediction. We have used Googles TensorFlow framework, in particular the Keras API of TensorFlow which allows for rapid prototyping of deep learning models, to implement our model and loss functions.

## Appendix: B. Additional Figures

In this appendix, we have included additional surveillance error grid plots (see Fig. 7) and prediction plots (see Fig. 8) for the four patients that are not presented in the results section.
